# Comprehensive ^18^F-FDG PET-based radiomics in elevating the pathological response to neoadjuvant immunochemotherapy for resectable stage III non-small-cell lung cancer: A pilot study

**DOI:** 10.3389/fimmu.2022.994917

**Published:** 2022-11-17

**Authors:** Yingpu Cui, Yaobin Lin, Zerui Zhao, Hao Long, Lie Zheng, Xiaoping Lin

**Affiliations:** ^1^ State Key Laboratory of Oncology in South China, Collaborative Innovation Center for Cancer Medicine, Sun Yat-sen University Cancer Center, Guangzhou, China; ^2^ Department of Nuclear Medicine, Sun Yat-Sen University Cancer Center, Guangzhou, China; ^3^ Department of Thoracic Surgery, Sun Yat-Sen University Cancer Center, Guangzhou, China; ^4^ Department of Radiology, Sun Yat-Sen University Cancer Center, Guangzhou, China

**Keywords:** non-small cell lung cancer, immunochemotherapy, ^18^F-FDG PET, pathological response, radiomics

## Abstract

**Purpose:**

To develop a comprehensive PET radiomics model to predict the pathological response after neoadjuvant toripalimab with chemotherapy in resectable stage III non-small-cell lung cancer (NSCLC) patients.

**Methods:**

Stage III NSCLC patients who received three cycles of neoadjuvant toripalimab with chemotherapy and underwent ^18^F-FDG PET/CT were enrolled. Baseline ^18^F-FDG PET/CT was performed before treatment, and preoperative ^18^F-FDG PET/CT was performed three weeks after the completion of neoadjuvant treatment. Surgical resection was performed 4–5 weeks after the completion of neoadjuvant treatment. Standardized uptake value (SUV) statistics features and radiomics features were derived from baseline and preoperative PET images. Delta features were derived. The radiologic response and metabolic response were assessed by iRECIST and iPERCIST, respectively. The correlations between PD-L1 expression, driver-gene status, peripheral blood biomarkers, and the pathological responses (complete pathological response [CPR]; major pathological response [MPR]) were assessed. Associations between PET features and pathological responses were evaluated by logistic regression.

**Results:**

Thirty patients underwent surgery and 29 of them performed preoperative PET/CT. Twenty patients achieved MPR and 16 of them achieved CPR. In univariate analysis, five SUV statistics features and two radiomics features were significantly associated with pathological responses. In multi-variate analysis, SUV_max_, SUV_peak_, SUL_peak_, and End-PET-GLDM-LargeDependenceHighGrayLevelEmphasis (End-GLDM-LDHGLE) were independently associated with CPR. SUV_peak_ and SUL_peak_ performed better than SUV_max_ and SUL_max_ for MPR prediction. No significant correlation, neither between the radiologic response and the pathological response, nor among PD-L1, driver gene status, and baseline PET features was found. Inflammatory response biomarkers by peripheral blood showed no difference in different treatment responses.

**Conclusion:**

The logistic regression model using comprehensive PET features contributed to predicting the pathological response after neoadjuvant toripalimab with chemotherapy in resectable stage III NSCLC patients.

## Introduction

Non–small cell lung cancer (NSCLC) is the leading cause of cancer-related death worldwide (1). Immunotherapy has become a new therapeutic approach in NSCLC and may provide prolonged benefit. Immune checkpoint inhibitors (ICIs), such as programmed cell death 1 (PD-1) and PD ligand 1 (PD-L1) monoclonal antibodies, have shown enormous survival benefits among patients with NSCLC (2). Toripalimab, a recombinant, humanized programmed death receptor-1 (PD-1) monoclonal antibody that binds to PD-1 and prevents binding of PD-1 with programmed death ligands 1 (PD-L1) and 2 (PD-L2), is being developed for the treatment of various cancers in China (3). Several phase 1/2 clinical trials of toripalimab have exhibited its manageable safety profile and promising antitumor activity among patients with various cancers, including melanoma, urothelial cancer, renal cell cancer, gastric cancer, and NSCLC (4).

Radiologic response assessment is important in treatment monitoring and clinical decision-making. The tumor response patterns of immunotherapy may be different from that of conventional therapies. The immunotherapy responses are variable and frequently delayed, complicating the evaluation of new immunotherapy agents and customizing treatment for individual patients. Lesions can initially increase in size due to an influx of immune cells during immunotherapy treatment, known as pseudo-progression (5, 6). Early anatomic imaging may show that a tumor has increased in size, but this could represent pseudo-progression. Conventional imaging criteria, either RECIST1.1 or iRECIST, have limitations depending on morphologic changes (5–7). ^18^F-Fluorodeoxyglucose positron emission tomography/computed tomography (^18^F-FDG PET/CT) has unique values in tumor staging, predicting prognosis, and evaluating treatment response. In addition, ^18^F-FDG PET/CT was considered to overcome limitations of anatomic imaging and more suitable for assessment of therapeutic effect, because it can reflect on tumor metabolic level before morphological changes (8).

The mechanism of ^18^F-FDG uptake within tumor cells is concerned with the presence of glucose metabolism, hypoxia, and angiogenesis (9–11). The mechanism of neoadjuvant immunotherapy is to induce a stronger and broader tumor-specific T-cell response when drug exposure occurs, which means that numerous immune cells consume glucose in the tumor microenvironment (TME) (8). It challenges to distinguish high uptake from the tumor cell from the immune cell by the classical metabolism parameters, such as standard uptake value (SUV), metabolic tumor volume (MTV), and total lesions glycolysis (TLG). Other imaging technologies and approaches are being developed to improve the measurement of response in patients receiving immunotherapy. Radiomics, as a widely recognized computational method for prognosis, exploits quantitative features (indicators) extracted from medical images to represent tumor characteristics (12). Through high-throughput feature extraction and statistical machine-learning methods, radiomics can extract and analyze tumor characteristics. It has drawn much attention among clinical oncologists due to its ability to provide comprehensive representations of tumor characteristics, including intra-tumor (13). Radiomics may provide additional information for pathological response prediction after neoadjuvant immunochemotherapy in stage III NSCLC patients.

The tumor is strongly associated with the microenvironment of tumor cells. Some studies found that tumor-associated inflammation plays a key part in tumor progression (14). Incorporation of the non-imaging metric into a multimodality efficacy metric may contribute to better-predicting tumor response, identifying resistance mechanisms, rationally selecting immune and other therapeutics, and ultimately improving patients’ lives. Tumor-associated inflammation could be quantified using several peripheral blood biomarkers and lymphoid organ metabolism on ^18^F-FDG PET and had a prognostic value of NSCLS after neoadjuvant treatment (15).

In our previous study, we reported that toripalimab plus platinum-based doublet chemotherapy yields a high major pathologic response (MPR) rate, manageable toxicity, and feasible resection in stage III NSCLC (3). The current study aims to evaluate the relationship between tumor metabolic parameters of ^18^F-FDG PET/CT, immune background biomarkers and the surgical pathology of these patients. Moreover, we try to develop a comprehensive PET radiomics-based model to predict the complete pathological response (CPR) and MPR after neoadjuvant toripalimab with chemotherapy in resectable stage III NSCLC patients.

## Materials and methods

### Design and patients

This phase 2 trial received Institutional Review Board (IRB) approval and was performed in a tertiary referral center using toripalimab, nab-paclitaxel or pemetrexed, and carboplatin in stage III NSCLC. Trial ClinicalTrials.gov (NCT04304248). Written informed consent was provided by all participants. The inclusion criteria were: 1) stage IIIA or T3-4N2 IIIB NSCLC according to the American Joint Committee on Cancer (AJCC) 8th edition; 2) no brain metastasis according to MRI; 3) surgically resectable; 4) age ≥ 18 years. 5) Eastern Cooperative Oncology Group (ECOG) performance status of 0 or 1. The exclusion criteria were: 1) EGFR exon 19/21 mutation or EML4-ALK translocation; 2) autoimmune disease or other conditions requiring immunosuppressive medicines within 14 days of enrollment. Patients received three cycles of neoadjuvant treatment (intravenous toripalimab, 240 mg, day 1; carboplatin, day 1; pemetrexed [500 mg/m2 for adenocarcinoma] or nab-paclitaxel [260 mg/m2 for other subtypes], day 1; 21-day each cycle). The cycles were determined before the trial started according to the previous studies (16, 17). Resection of the primary tumor and ipsilateral lymph nodes would be performed 4–5 weeks after the first dose for patients with no progression according to the radiographic evaluation. Adjuvant toripalimab monotherapy or other adjuvant modalities determined by the multidisciplinary team were performed from 4–8 weeks after surgery to 12 months. Chest CT has been performed every 3 months during the first two years and every 6 months afterward following surgery. The protocol has been published elsewhere (3).

### ^18^F-FDG PET/CT imaging

Baseline ^18^F-FDG PET/CT was performed for 13 patients and preoperative ^18^F-FDG PET/CT was performed for 29 patients. Preoperative PET/CT was performed three weeks after the completion of neoadjuvant treatment. Images were archived from the integrated PET/CT scanner (Biograph mCT, Siemens Healthcare; uEXPLORER, United Imaging Healthcare). All patients fasted for 5 to 6 hours and make sure their blood glucose levels were lower than 11.1 mmol/L. ^18^F-FDG (3.7 ± 0.37 MBq [0.1 ± 0.01 mCi]/kg) were administered intravenously. After 60 ± 10 min, PET/CT was scanned. According to the guidelines of the European Association of Nuclear Medicine (18), the PET was scanned 2min/bed with bed overlap > 30%. The PET images obtained from biograph mCT were reconstructed using the ordered subsets expectation maximization (OSEM), with a slice thickness of 2 mm (3D) in a 200×200 matrix. The PET images obtained from uEXPLORER were reconstructed using the ordered subset expectation maximization (OSEM) algorithm with the following parameters: time of flight (TOF) and point spread function (PSF) modeling, 3 iterations and 20 subsets, matrix 256 × 256, slice thickness of 2.886 mm and the Gaussian filter function 3 mm. No significant difference in the liver and aorta metabolic parameters was found between these two scanners (**Supplementary Material 1**).

### Image annotation and interpretation

We loaded PET/CT images to 3D slicer software (version 4.8.0; http://www.slicer.org). Lung lesions were annotated on the baseline and preoperative PET by the two junior nuclear medicine physicians (with 3 years of experience) in a blinded fashion. All annotations were validated by a senior nuclear medicine physician. If there were no lung lesions after treatment, adjacent normal or atelectasis lung tissue was annotated instead. The interobserver reproducibility was evaluated using the intraclass correlation coefficient (ICC) for radiomics features derived from the annotation. Features with ICC > 0.75 were selected. Lymph nodes were not annotated in this analysis.

Furthermore, six SUV statistics PET features were extracted from the original images: maximal SUV (SUV_max_), peak SUV (SUV_peak_), maximal SUV normalized for lean body mass (SUL_max_), peak SUL (SUL_peak_), MTV, and TLG. SUV_peak_ is defined as the average SUV of a 1 cm diameter ball centered on the maximum intensity pixel of the tumor. MTV is calculated using the threshold of 41% of the SUV_max_. TLG is calculated using the formula TLG=MTV x SUV_mean_. Lesion-to-liver SUV_max_ ratio (LLR) was calculated for preoperative PET. The Deauville 5-point scale (5PS) was also assessed, from a Deauville score (DS) of 1 to 5.

FDG PET inflammatory parameters were also enrolled, including bone marrow to liver ratio (BLR) and spleen to liver ratio (SLR), calculated by dividing the bone marrow (BM) SUV_max_ by the liver SUV_max_ and the spleen SUV_max_ by the liver SUV_max_, respectively. As published in several studies (15, 19, 20), liver, spleen, and BM SUV_max_ values were calculated by drawing a spherical 3 cm-volume of interest (VOI) in the right upper lobe of the liver, a spherical 2 cm-VOI in the spleen, and 4 spherical 1.5 cm-VOI in the center of L1 to L4 (lumbar) vertebral bodies for BM. BM SUV_max_ was calculated by averaging SUV_max_ values of all four vertebral VOIs. All VOIs avoided the edge and metastatic lesions.

All patient radiologic responses were assessed according to iRECIST 1.1. For patients with baseline PET/CT and preoperative PET/CT, responses were also assessed using iPERCIST. In iPERCIST, response to the neoadjuvant therapy was classified as (1) CMR, defined as the disappearance of any uptake in the target lesion; (2) partial metabolic response (PMR), defined as a reduction of 30% or more in the target tumor SUL_peak_; (3) progressive metabolic disease (PMD), defined as 30% or more increase in SUL_peak_ or new metabolically active lesions: immune unconfirmed PMD (iuPMD); (4) stable metabolic disease (SMD), defined as disease other than CMR, PMR, or PMD; (5) Confirmed PMD (cPMD): reevaluation 4-8 weeks later to diagnose cPMD.

### Radiomics feature extraction

**Figure 1:**
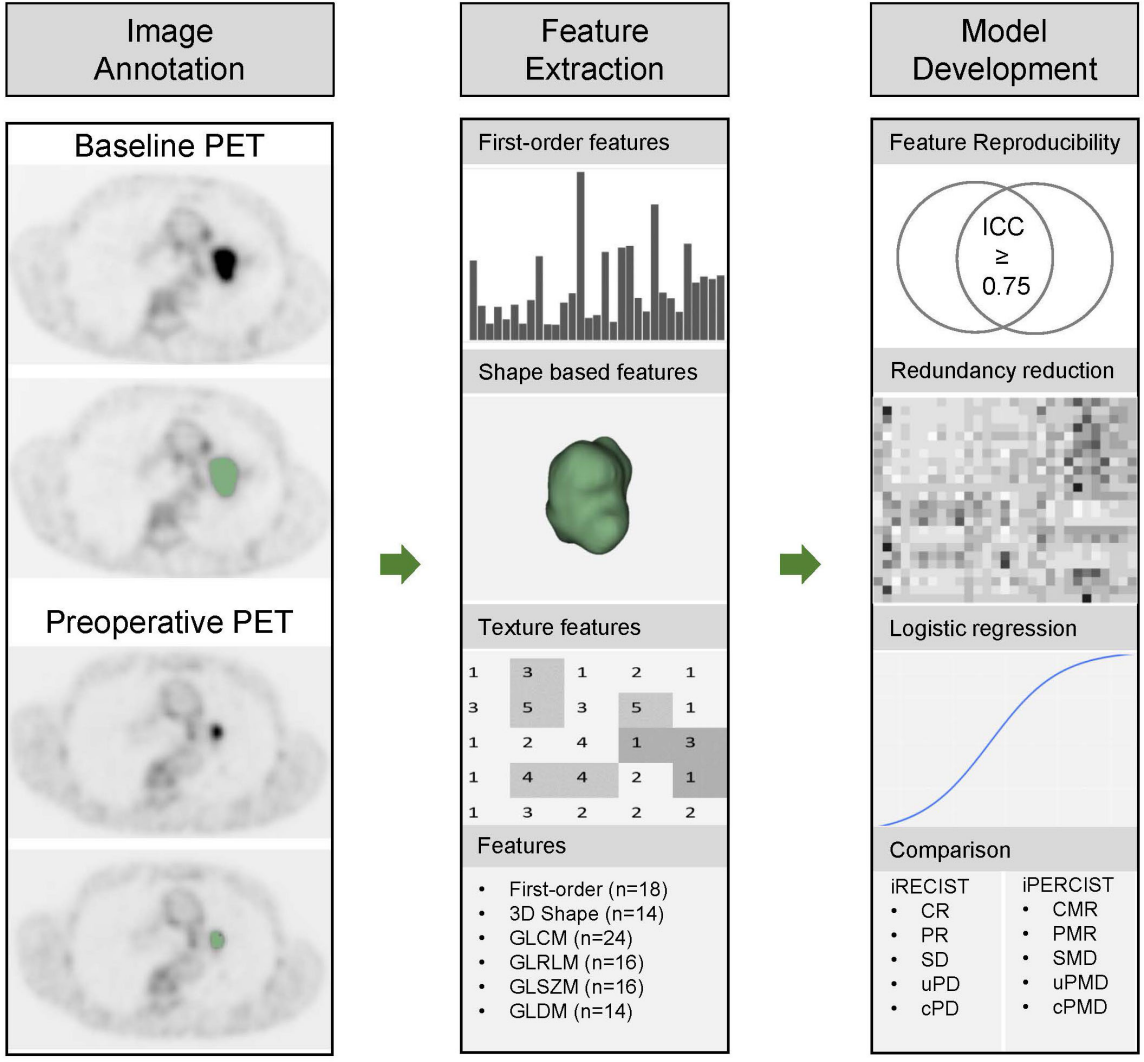
Workflow for pathological responses prediction and comparison with iRECIST and iPERCIST. The workflow includes image annotation, feature extraction, and model development. 3D, three dimension; GLCM, gray level co-occurrence matrix; GLDM, gray level dependence matrix; GLRLM, gray level run length matrix; GLSZM, gray level size zone matrix; ICC, intraclass correlation coefficient.

Radiomics features were extracted from the PET scans and the corresponding 3D annotation using the Python package Pyradiomics (12), which complies with the Image Biomarker Standardization Initiative (IBSI) (13). Feature extraction parameters were set as a specific value (binWidth: 0.25; interpolator: sitkBSpline; resampledPixelSpacing: None) (21, 22). 102 radiomics features of 6 categories were extracted as follows: the shape, histogram (first-order statistics), gray level co-occurrence matrix (GLCM), gray level dependence matrix (GLDM), gray level run length matrix (GLRLM), and gray level size zone matrix (GLSZM) features. The list of the extracted radiomics features was shown in **Table 1**. The workflow was shown in **Figure 1**.

**Table 1:** The list of extracted radiomics features.

First-order statistics (n=18)	Shape-based (n=14)	GLCM (n=24)	GLRLM (n=16)	GLSZM (n=16)	GLDM (n=14)
10^th^ Percentile	Elongation	Autocorrelation	GLN	GLN	DE
90^th^ Percentile	Flatness	Cluster Prominence	GLNN	GLNN	DN
Energy	Least Axis Length	Cluster Shade	GLV	GLV	DNN
Entropy	Major Axis Length	Cluster Tendency	HGLRE	HGLZE	DV
Interquartile Range	Maximum 2D Diameter (Column)	Contrast	LRE	LAE	GLN
Kurtosis	Maximum 2D Diameter (Row)	Correlation	LRHGLE	LAHGLE	GLV
Maximum	Maximum 2D Diameter (Slice)	Difference Average	LRLGLE	LALGLE	HGLE
MAD	Maximum 3D Diameter	Difference Entropy	LGLRE	LGLZE	LDE
rMAD	Mesh Volume	Difference Variance	RE	SZN	LDHGLE
Mean	Minor Axis Length	ID	RLN	SZNN	LDLGLE
Median	Sphericity	IDM	RLNN	SAE	LGLE
Minimum	Surface Area	IDMN	RP	SAHGLE	SDE
Range	Surface Volume Ratio	IDN	RV	SALGLE	SDHGLE
RMS	Voxel Volume	IMC1	SRE	ZE	SDLGLE
Skewness		IMC2	SRHGLE	ZP	
Total Energy		Inverse Variance	SRLGLE	ZV	
Uniformity		Joint Average			
Variance		Joint Energy			
		Joint Entropy			
		MCC			
		Maximum Probability			
		Sum Average			
		Sum Entropy			
		Sum of Squares			

### Feature redundancy reduction

Six SUV statistics features (SUV_max_, SUV_peak_, SUL_max_, SUL_peak_, MTV, TLG), 102 radiomics features (including SUV_max_), and two FDG PET inflammatory parameters (BLR, SLR) were put together. Pearson Correlation Coefficient (PCC) was applied to calculate the collinearity of each feature pair. If the PCC value of the feature pair was larger than 0.700, one of the features was removed. Considering the wide application of SUV statistics features in clinical practice, when collinearity was observed between any SUV statistic features and radiomics features, SUV statistics features were retained.

### Clinical analyses

The pathological assessment was performed (23). For tumor bed samples < 6 cm, the entire tumor bed samples were submitted. For tumor bed samples ≥ 6 cm, the samples were assessed at least one section/cm along the major axis. The pathologic assessment procedure was detailed in **Supplementary Material 2**. MPR was defined as a 10% or less viable residual tumor in the resected specimen. CPR was defined as no viable tumor on all slides of the entire tumor bed.

Exploratory analyses, including the PD-L1 expression assessment and next-generation sequencing (NGS), are described in **Supplementary Material 2**.

Complete blood cell counts, lactate dehydrogenase (LDH), and CRP tests were performed within two weeks before the first cycle and more than three weeks after the completion of neoadjuvant treatment. The peripheral blood inflammatory biomarkers included serum LDH, C-reactive protein (CRP), derived neutrophils/(leucocytes-neutrophils) ratio (dNLR), platelet to lymphocyte ratio (PLR) and systemic inflammation index (SII). Indices were derived using the formulas: dNLR = neutrophil/(leukocyte - neutrophil), PLR = platelet/lymphocyte, and SII = platelet × neutrophil/lymphocyte. Cutoffs for LDH, CRP, dNLR, PLR, and SII were set: high LDH if > 245 IU/L, high CRP if ≥ 50 mg/L (24), high dNLR if > 3 (25), high PLR if≥ 150 (26) and high SII if ≥ 1,270 (27), from the largest available studies of advanced NSCLCs treated with chemotherapy and/or immunotherapy.

### Model development

The endpoint was the pathological response (CPR or MPR) of the lung tumor. The associations between PET parameters and pathological response were assessed by Logistic regression. Initially, the separate association between each parameter and outcome was assessed in a series of univariable logistic analyses. Features with *p* values <0.05 were selected. Subsequently, the joint association between the selected variables was assessed using multivariate logistic regression with backward selection.

### Statistical analysis

Continuous variables were summarized as median (interquartile range), and categorical variables were summarized using counts (percentage). The Mann-Whitney U test, χ2 test, or Fisher exact test was used to compare the features between CPR and Non-CPR, MPR, and Non-MPR subgroups accordingly. The correlations between the radiologic or metabolic response and pathological response were assessed using the Mann-Whitney U test. The correlations between the baseline PET features and PD-L1 expression or driver-gene status were assessed using the Mann-Whitney U test. We computed the data using programming languages (python version 3.5.0, Python Software Foundation, https://www.python.org; R version 4.0.0, Comprehensive R Archive Network, https://www.r-project.org) and statistical software (IBM SPSS Statistics version 15.0 for Windows).

## Results

### Patient characteristics

**Figure 2:**
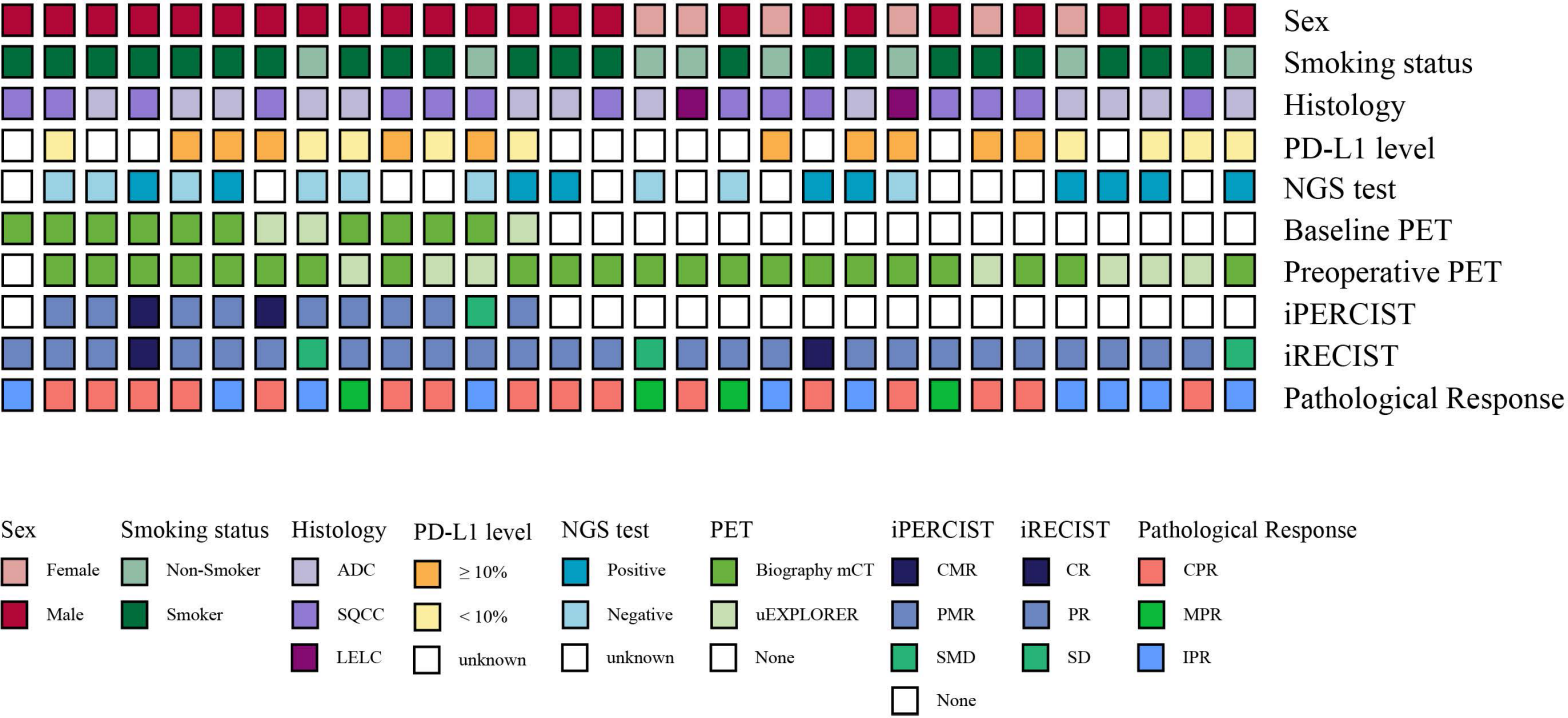
Clinical characteristics, radiological responses, metabolic responses, and pathological responses of the enrolled patients. Each bar represents one patient.

pt?>Thirty patients underwent surgery and 29 of them performed preoperative PET/CT. The characteristics of the 30 patients are summarized in **Table 2**. 15 (50.0%) had squamous cell carcinoma (SQCC), 13 (42.9%) had adenocarcinoma, and 2 (7.1%) had lymphoid epithelial-like carcinoma (LELC). For patients with LELC, an endoscopic examination of the nasopharynx was conducted to rule out metastatic LELC from the nasopharynx. 18 (60.0%) patients had stage IIIA disease, and 12 (40.0%) had stage IIIB disease. 20 (66.7%; 95% CI 47.2–82.7) achieved MPR, of whom 16 (53.3%; 34.3–71.7) achieved CPR. Thirteen patients underwent baseline PET/CT and 29 did preoperative PET/CT, including 12 who did both baseline and preoperative (paired) PET/CT (**Figure 2**). There was no significant difference in age, gender, smoking status, baseline clinical stage (IIIA or IIIB), and baseline, preoperative, and paired PET distribution between CPR and Non-CPR, MPR and Non-MPR subgroups (all *p* > 0.05) (**Table 2**). No difference was found between adenocarcinoma and SQCC in terms of pathological response (*p*=0.128 for CPR vs. Non-CPR; *p*=0.114 for MPR vs. Non-MPR). The distributions of six SUV statistics features (SUV_max_, SUV_peak_, SUL_max_, SUL_peak_, MTV, and TLG) of baseline, preoperative, and delta PET between different groups were summarized in **Supplementary Material 1** and **Table 3**.

**Table 2:** General information between patients achieved complete pathological response (CPR) and Non-CPR, major pathological response (MPR) and Non-MPR.

Characteristic	CPR (n=16)	Non-CPR (n=14)		*p*	MPR (n=20)	Non-MPR (n=10)	*p*
Age, median (range)	59 (33-71)		60 (35-67)	0.313	60 (33-71)	58 (35-64)	0.183
Sex				1.000			1.000
Female	3		3		4	2	
Male	13		11		16	8	
Histology				–			–
Adencarcioma	4		9		6	7	
Squamous cell cancer	10		5		12	3	
Lymphoepithelioma like carcinoma	2		0		2	0	
Stage				0.296			0.461
IIIA	11		7		13	5	
IIIB	5		7		7	5	
Smoking				0.101			0.078
No	2		6		3	5	
Yes	14		8		17	5	
Baseline PET				0.484			1.000
No	8		9		11	6	
Yes	8		5		9	4	
Preoperative PET				0.467			0.333
No	0		1		0	1	
Yes	16		13		20	9	
Paired PET				0.284			0.694
No	8		10		11	7	
Yes	8		4		9	3	

**Table 3:** The distributions of selected baseline, delta, and preoperative PET features and the univariate logistic regression results for preoperative PET features.

feature	CPR	Non-CPR	*p* value	logistic *p* value	MPR	Non-MPR	*p* value	logistic *p* value
Baseline-SUV_max_	13.355 (11.449, 19.961)	13.768 (12.954, 20.337)	0.622	–	13.487 (11.997, 20.337)	13.361 (11.941, 19.570)	0.940	–
Baseline-SUV_peak_	11.115 (9.725, 16.730)	11.590 (11.530, 15.540)	0.833	–	11.800 (10.330, 15.640)	11.560 (9.730, 16.680)	1.000	–
Baseline-SUL_max_	10.781 (9.334, 14.589)	9.873 (9.773, 16.731)	0.833	–	11.079 (9.536, 10.062)	9.823 (9.161, 16.002)	0.825	–
Baseline-SUL_peak_	8.956 (7.920, 12.680)	8.699 (8.311, 12.785)	0.943	–	9.172 (7.979, 12.785)	8.505 (7.257, 13.844)	0.825	–
Baseline-MTV	19.707 (13.569, 33.120)	12.872 (8.460, 15.711)	0.127	–	19.541 (10.948, 28.916)	14.292 (10.666, 17.394)	0.414	–
Baseline-TLG	153.913 (97.984, 413.084)	122.952 (96.115, 150.994)	0.524	–	135.785 (96.115, 402.209)	136.973 (87.855, 178.239)	0.825	–
Delta-SUV_max_	0.722 (0.681-0.799)	0.339 (0.241-0.393)	0.028	–	0.69 (0.677-0.784)	0.334 (0.149-0.339)	0.036	–
Delta-SUV_peak_	0.759 (0.632-0.817)	0.407 (0.264-0.526)	0.016	–	0.746 (0.619-0.788)	0.381 (0.264-0.407)	0.009	–
Delta-SUL_max_	0.734 (0.686-0.798)	0.347 (0.244-0.403)	0.028	–	0.701 (0.679-0.784)	0.338 (0.15-0.347)	0.036	–
Delta-SUL_peak_	0.764 (0.643-0.815)	0.415 (0.266-0.533)	0.016	–	0.745 (0.622-0.788)	0.385 (0.266-0.415)	0.009	–
Delta-MTV	0.915 (0.871, 0.959)	0.755 (0.367, 0.789)	0.028	–	0.915 (0.866, 0.958)	0.783 (0.395, 0.789)	0.100	–
Delta-TLG	0.966 (0.942-0.99)	0.832 (0.696-0.861)	0.004	–	0.965 (0.939-0.99)	0.807 (0.585-0.832)	0.009	–
Delta-GLDM-DN	0.960 (0.914-0.985)	0.668 (0.561-0.761)	0.008	–	0.946 (0.909-0.985)	0.581 (0.542-0.668)	0.009	–
End-SUV_max_	3.081 (2.165-4.172)	6.397 (4.646-9.347)	0.001	0.011	3.364 (2.165-4.31)	8.315 (6.302-9.56)	<0.001	0.007
End-SUV_peak_	2.525 (1.645-3.020)	5.180 (3.650-7.170)	0.001	0.008	2.555 (1.678-3.275)	5.380 (5.150-7.200)	<0.001	0.010
End-SUL_max_	2.332 (1.564-3.053)	4.781 (3.653-6.536)	0.003	0.015	2.369 (1.564-3.399)	6.338 (4.745-6.536)	<0.001	0.009
End-SUL_peak_	1.883 (1.162-2.354)	3.948 (2.870-4.889)	0.002	0.009	1.883 (1.162-2.426)	4.082 (3.641-5.113)	<0.001	0.008
End-MTV	0.917 (0.552-1.874)	3.916 (2.389-11.319)	0.001	0.284	1.292 (0.564-2.397)	4.147 (3.815-11.319)	0.006	0.393
End-TLG	2.803 (0.826-6.239)	20.948 (12.496-29.184)	<0.001	0.046	3.734 (0.880-8.951)	29.149 (20.948-42.3)	<0.001	0.022
End-Uniformity	0.215 (0.134-0.399)	0.104 (0.072-0.149)	0.007	0.046	0.198 (0.134-0.352)	0.091 (0.072-0.134)	0.003	0.039
End-GLDM-LDHGLE	124.211 (84.558-408.893)	782.833 (537.296-1032.684)	0.001	0.007	257.708 (103.544-589.779)	783.159 (718.023-1032.684)	0.005	0.027

### Peripheral blood biomarkers and pathological response

In 30 patients who underwent surgery, 28 had baseline LDH and CRP tests, 30 had baseline peripheral blood tests, 30 had preoperative LDH and CPR tests, and 29 had preoperative peripheral blood tests. There was no significant difference in the LDH, CRP, dNLR, PLR, and SII of either baseline or preoperative or delta, between CPR and Non-CPR, MPR and Non-MPR subgroups (all *p* > 0.05) (**Supplementary Material 3**).

No significant correlation was found between the age, gender, smoking status, histology, or baseline clinical stage and LDH, CRP, dNLR, PLR, or SII (all *p* > 0.05). No significant correlation was found between BLR, SLR, or other PET features and LDH, CRP, dNLR, PLR, or SII, either in baseline or preoperative examinations (all *p* > 0.05) (**Supplementary Material 3**).

### Clinical biomarkers and pathological response

Of 30 patients who underwent surgery, 19 patients underwent the PD-L1 test. The percentage of patients who achieved an MPR with positive PD-L1 expression was similar to that of those with negative PD-L1 expression. The results had been published elsewhere (3). Ten of 18 patients underwent baseline PET, and the distributions of features were not significant between the positive and negative PD-L1 expression group. In 30 patients who underwent surgery, 19 patients underwent NGS, and all of them had negative EGFR and EML4-ALK results from baseline core biopsy specimens (amplification-refractory mutation system for EGFR mutation screening and immunohistochemistry for EML4-ALK rearrangement testing). However, four had positive NGS results from postoperative specimens (two had EGFR exon 19 del, one had EGFR L858R, and one had focal [5%] ALK positivity). None of them achieved MPR. The results had been published elsewhere (3). Nine of 19 patients underwent baseline PET. In specific gene mutations, such as STK11, KEAP1, RB1, and EGFR, as the driver-gene positive group, the distribution of features was not significant between the positive and the negative group.

### Radiologic response, metabolic response, and pathological response

According to RECIST, in 30 patients who had undergone thoracic tumor resection, two achieved complete response (CR), 25 achieved partial response (PR), and three patients achieved stable disease (SD). No significant correlation between the radiologic response and pathological response was seen.

**Figure 3:**
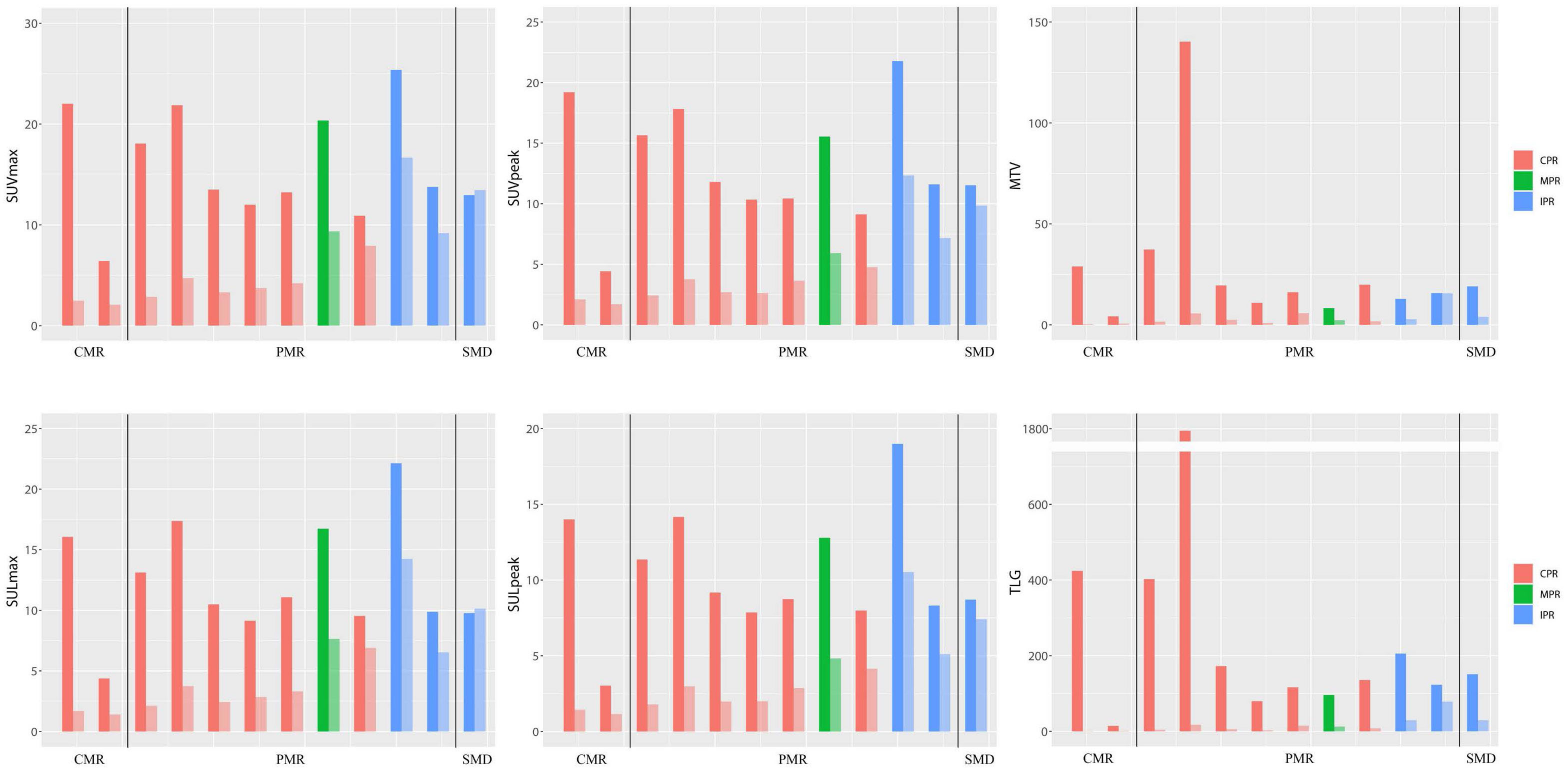
Bar plot for six SUV statistics features of paired PET (baseline and preoperative). The dark colors represent baseline PET features, and the light colors represent preoperative PET features. The patient number from left to right is 16, 5, 21, 23, 4, 12, 20, 19, 1, 14, 18, and 22, respectively. CMR, complete metabolic response; PMR, partial metabolic response; SMD, stable metabolic disease; CPR, complete pathological response; MPR, major pathological response; IPR, incomplete pathological response.

**Figure 4:**
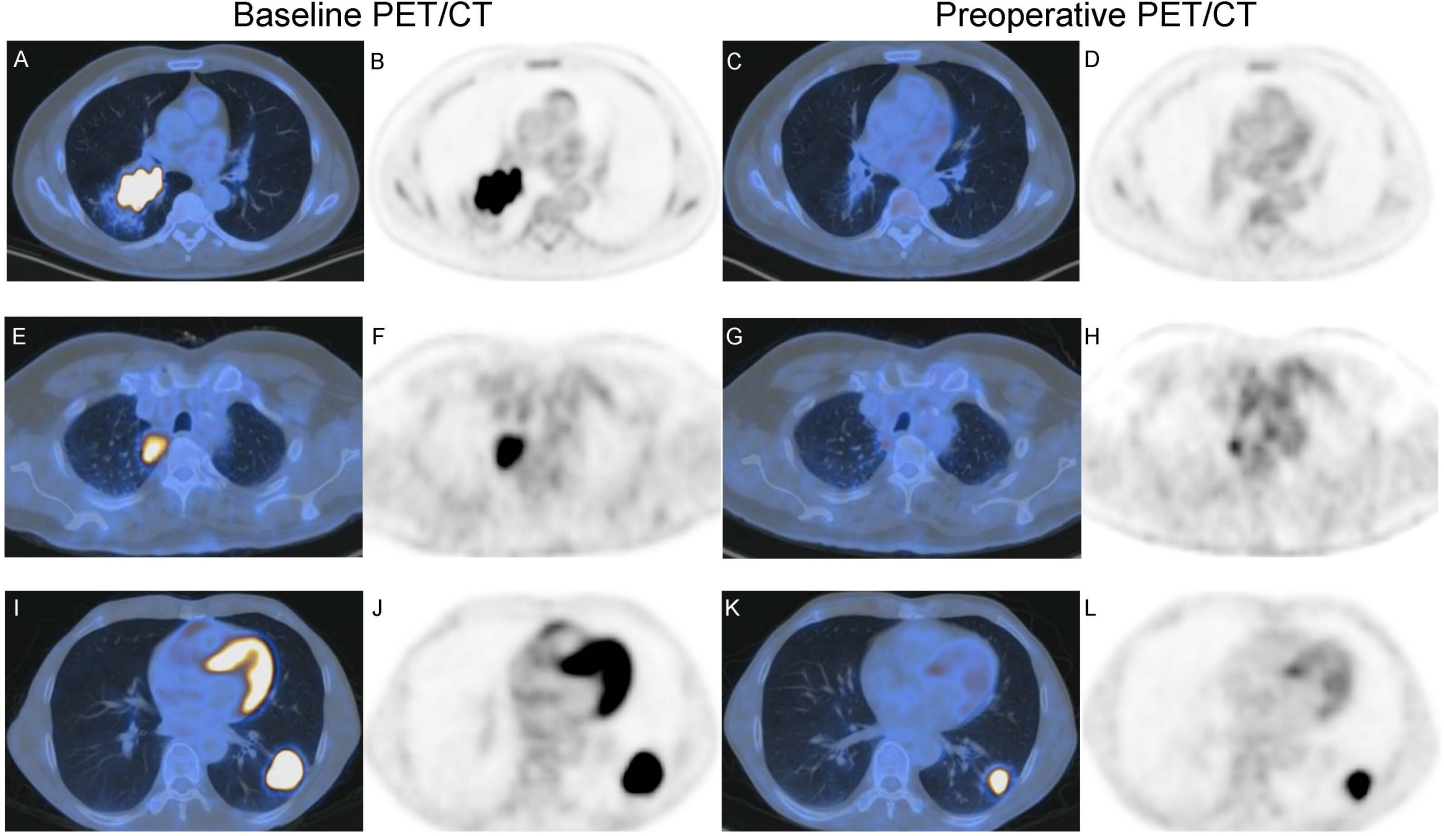
Baseline imaging and preoperative PET/CT of two patients with NSCLC receiving neoadjuvant toripalimab with chemotherapy. **(A–D)** A 58-year-old male with squamous cell lung cancer (SQCC) had CMR according to iPERCIST. **(A, B)** show baseline axial fused image and PET, SUL_peak_ = 14.0. **(C, D)** show preoperative axial fused image and PET, SUL_peak_ = 1.44. Resection specimen showed this patient had CPR, consistent with preoperative PET. **(E–H)** A 65-year-olf male with adenocarcinoma had PMR according to iPERCIST. **(E, F)** show baseline axial fused image and PET, SUL_peak_ = 7.86. **(G, H)** show preoperative axial fused image and PET, SUL_peak_ = 2.00. Resection specimen showed this patient had CPR, inconsistent with preoperative PET. **(I–L)** A 52-year-olf male with adenocarcinoma had PMR according to iPERCIST. **(I, J)** show baseline axial fused image and PET, SUL_peak_ = 19.0. **(K, L)** show preoperative axial fused image and PET, SUL_peak_ = 10.5. Resection specimen showed this patient had IPR, consistent with preoperative PET.

According to iPERCIST, of 12 patients who underwent the baseline, preoperative PET/CT, and surgery, two patients achieved CMR, nine achieved PMR, and one achieved SMD (**Table 4** and **Figure 3**). There was no significant correlation between the metabolic and pathological response. Cases with baseline imaging and preoperative PET/CT were shown in **Figure 4**.

**Table 4:** Association between radiologic response, metabolic response, and pathological response.

	CPR (n=16)	Non-CPR (n=14)	*p*	MPR (n=20)	Non-MPR (n=10)	*p*
iRECIST			0.154			0.328
CR	2	0		2	0	
PR	14	11		17	8	
SD	0	3		1	2	
	CPR (n=8)	Non-CPR (n=4)	*p*	MPR (n=9)	Non-MPR (n=3)	*p*
iPERCIST			0.283			0.282
CMR	2	0		2	0	
PMR	6	3		7	2	
SMD	0	1		0	1	

### Radiomics features and pathological response

In 102 radiomics features (including SUV_max_), 76 features were highly reproducible with ICCs higher than 0.75. 76 features and six SUV statistics features (SUV_max_, SUV_peak_, SUL_max_, SUL_peak_, MTV, and TLG) were included for further analysis. Pearson Correlation Coefficient (PCC) was applied to reduce feature redundancy. Baseline PET features, preoperative PET features, and delta PET features calculated from the forum (delta = [baseline – preoperative]/baseline) were analyzed. After this step, two SUV statistics features (Baseline-SUV_max_, Baseline-TLG), three baseline radiomics features, and FDG PET inflammatory features (Baseline-BLR, Baseline-PLR) were reserved for baseline PET. Two SUV statistics features (Delta-SUV_max_, Delta-TLG), six delta radiomics features, and FDG PET inflammatory features (Delta-BLR, Delta-PLR) were reserved for delta PET. Two SUV statistics features (End-SUV_max_, End-TLG), five preoperative radiomics features, and two FDG PET inflammatory features (End-BLR, End-PLR) were reserved for preoperative PET. Although the PCC value among SUV_max_, SUV_peak_, SUL_max_, and SUL_peak_ > 0.70, and the PCC value between TLG and MTV > 0.70, six SUV statistics features (SUV_max_, SUV_peak_, SUL_max_, SUL_peak_, MTV, and TLG) were all included to compare their performance.

**Figure 5:**
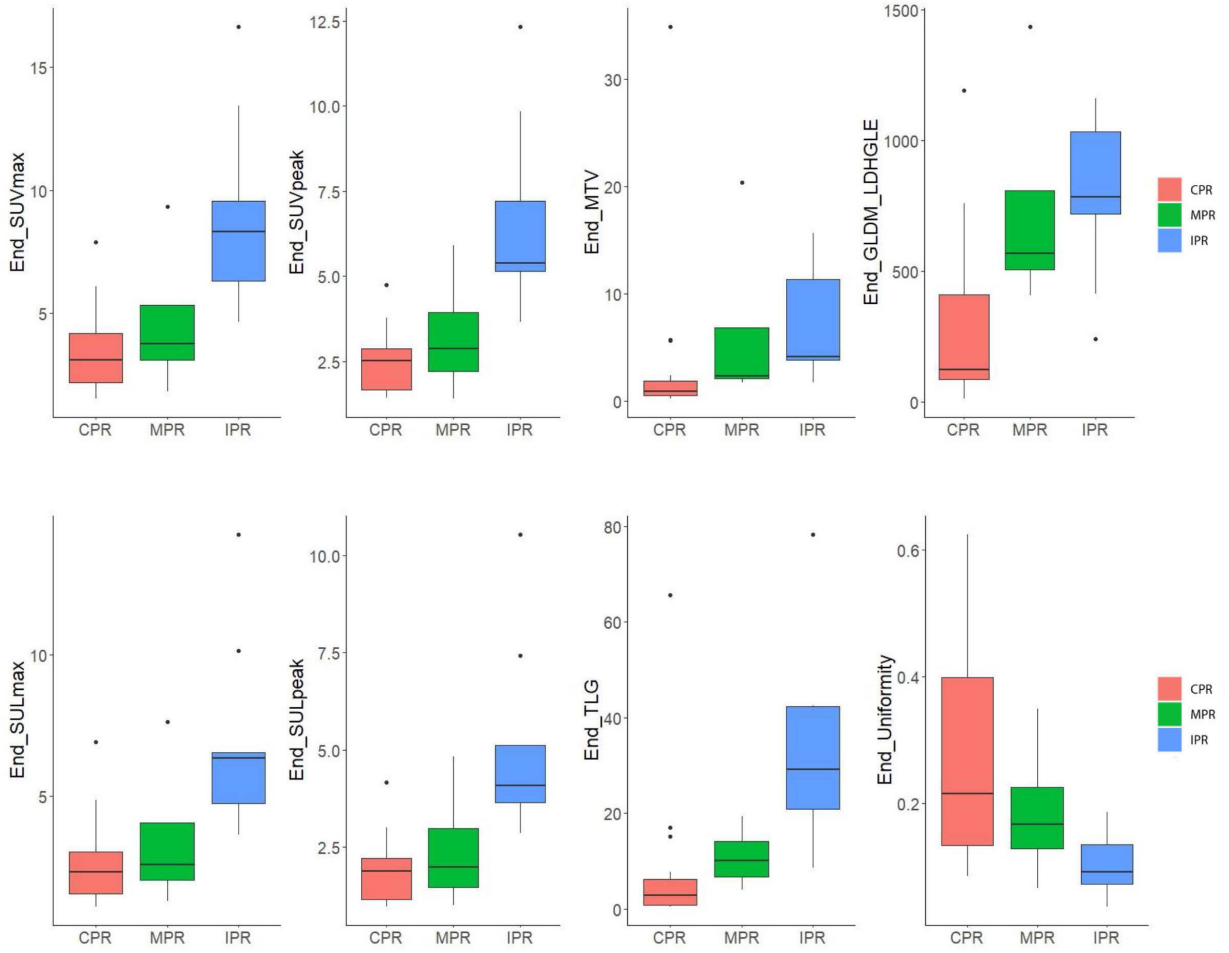
Box plot for six SUV statistics features and two radiomics features of preoperative PET of 29 patients. The red box represents the complete pathological response (CPR) group. The green box represents the major pathological response (CPR) group. The blue box represents the incomplete pathological response (IPR) group. Lengths of whiskers are limited to a maximum of 1.5 times the interquartile range. Box indicates the interquartile range around the median.

For preoperative PET features, six SUV statistics features (SUV_max_, SUV_peak_, SUL_max_, SUL_peak_, MTV, and TLG) and two radiomics features (End-original-firstorder-Uniformity [End-Uniformity],End-original-GLDM-LargeDependenceHighGrayLevelEmphasis [End-GLDM-LDHGLE]) distributions were significantly different both in CPR vs. Non-CPR and MPR vs. Non-MPR groups (**Figure 5**). For delta PET features, five SUV statistics features (SUV_max_, SUV_peak_, SUL_max_, SUL_peak_, and TLG) and one radiomics feature (Delta-original-GLDM-DependenceNonUniformity [Delta-GLDM-DN]) distributions significantly differed both in CPR and Non-CPR, MPR and Non-MPR subgroups. The distributions of six SUV statistics features of paired PET were shown in **Figure 3**. No baseline PET feature distribution was significantly different either in the CPR and Non-CPR or the MPR and Non-MPR subgroups. The distributions of features between different groups were summarized in **Table 3**.

### Logistic model construction

**Figure 6:**
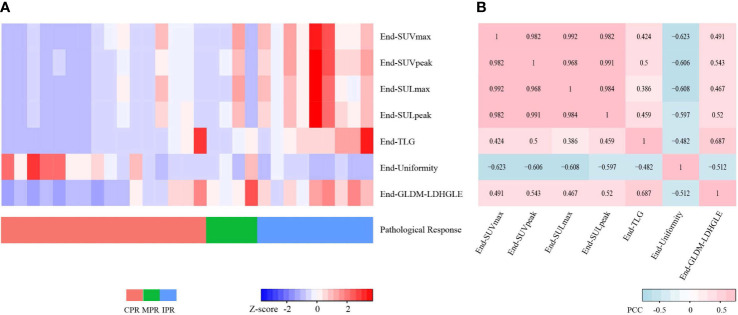
Heatmaps for seven preoperative PET features. **(A)** Heatmap shows the Z-scores of the seven preoperative PET features of 29 cases and corresponding pathological responses. **(B)** Heatmap shows the Pearson Correlation Coefficient (PCC) values of the seven preoperative PET features. CPR, complete pathological response; MPR, major pathological response; IPR, incomplete pathological response.

For preoperative PET features, the univariate logistic regression identified the seven most independent factors to predict CPR and MPR, including five SUV statistics features (SUV_max_, SUV_peak_, SUL_max_, SUL_peak_, and TLG) and two radiomics features (End-Uniformity, End-GLDM-LDHGLE) (**Table 3**). **Figure 5** illustrated the distribution of the selected features. The PCC values of these features were shown in **Figure 6**.

Considering the collinearity of SUV_max_, SUV_peak_, SUL_max,_ and SUL_peak_, each one of them combining TLG and two radiomics features was included for the further multivariate logistic analysis. The multivariate logistic analysis suggested that SUV_max_, SUV_peak_, SUL_peak_, and End-GLDM-LDHGLE were independently associated with CPR. SUV_max_, SUV_peak_, SUL_max_, and SUL_peak_ were independently associated with MPR. The detailed information on the constructed multivariate logistic models was shown in **Table 5** and **Supplementary Material 4**.

**Table 5:** Multi-variate results for complete pathological response (CPR) and major pathological response (MPR) prediction with preoperative PET features.

Multi-variate logistic model	Variable			Model
	SUV features	End-GLDM-LDHGLE	TLG
CPR				
SUV_max_	0.037	0.042	–	<0.001
SUV_peak_	0.027	0.074	–	<0.001
SUL_max_	0.057	0.037	–	<0.001
SUL_peak_	0.036	0.061	–	<0.001
MPR				
SUV_max_	0.025	–	0.073	<0.001
SUV_peak_	0.010	–	–	<0.001
SUL_max_	0.033	–	0.062	<0.001
SUL_peak_	0.008	–	–	<0.001

### LLR, 5-point Deauville scale (5PS), and pathological response

For preoperative PET, LLR achieved AUCs of 0.803 and 0.872, slightly inferior to those of SUV_max_ (0.851 and 0.911) for CPR and MPR prediction, respectively. The optimal cutoff value of LLR was 1.50. The sensitivity, specificity, and accuracy were 69.2, 87.5, and 79.3 for CPR, and 88.9, 85.0, and 86.2 for MPR, respectively. The accuracies were equal to those of SUV_max_. The 5PS was assessed. Coincidentally, the cases assessed as DS 5 were identical with LLR ≥ 1.50. Applying DS = 4 as a cutoff value, the sensitivity, specificity, and accuracy were 92.3, 50.0, and 69.0 for CPR, and 100.0, 45.0, and 62.1 for MPR, respectively. The detailed information was shown in **Table 6**.

**Table 6:** Discrimination for predicting pathological response using different criteria.

	SUV_max_	SUV_peak_	SUL_max_	SUL_peak_	End-GLDM-LDHGLE	TLG	LLR	5PS
CPR								
AUC	0.851 (0.670-0.955)	0.851 (0.670-0.955)	0.817 (0.630-0.935)	0.827 (0.641-0.941)	0.861 (0.682-0.960)	0.880 (0.705-0.970)	0.803 (0.614-0.926)	0.832 (0.647-0.944)
Sensitivity	76.9 (46.2-95.0)	76.9 (46.2-95.0)	76.9 (46.2-95.0)	69.2 (38.6-90.9)	92.3 (64.0-99.8)	100.0 (75.3-100.0)	69.2 (38.6-90.9)	92.3 (64.0-99.8)
Specificity	81.3 (54.4-96.0)	87.5 (61.7-98.4)	81.3 (54.4-96.0)	93.8 (69.8-99.8)	75.0 (47.6-92.7)	68.8 (41.3-89.0)	87.5 (61.7-98.4)	50.0 (24.7-75.3)
Accuracy	79.3 (60.3-92.0)	82.8 (64.2-94.2)	79.3 (60.3-92.0)	82.8 (64.2-94.2)	82.8 (64.2-94.2)	82.8 (64.2-94.2)	79.3 (60.3-92.0)	69.0 (49.2-84.7)
Threshold	4.20	3.65	3.32	3.00	349.5	3.92	1.50	4
MPR								
AUC	0.928 (0.768-0.990)	0.956 (0.807-0.998)	0.894 (0.724-0.977)	0.933 (0.776-0.992)	0.822 (0.636-0.938)	0.917 (0.753-0.986)	0.889 (0.717-0.975)	0.894 (0.724-0.977)
Sensitivity	100.0 (66.4-100.0)	100.0 (66.4-100.0)	100.0 (66.4-100.0)	100.0 (66.4-100.0)	77.8 (40.0-97.2)	100.0 (66.4-100.0)	88.9 (51.8-99.7)	100.0 (66.4-100.0)
Specificity	80.0 (56.3-94.3)	85.0 (62.1-96.8)	80.0 (56.3-94.3)	80.0 (56.3-94.3)	85.0 (62.1-96.8)	75.0 (50.9-91.3)	85.0 (62.1-96.8)	45.0 (23.1-68.5)
Accuracy	86.2 (68.3-96.1)	89.7 (72.6-97.8)	86.2 (68.3-96.1)	86.2 (68.3-96.1)	82.8 (64.2-94.2)	82.8 (64.2-94.2)	86.2 (68.3-96.1)	62.1 (42.3-79.3)
Threshold	4.20	3.65	3.32	2.64	674.3	7.77	1.50	4

## Discussion

In this study, we developed a logistic regression model using comprehensive PET features to predict the pathological response after neoadjuvant toripalimab plus chemotherapy treatment in patients with stage III NSCLCs. The proposed models based on preoperative PET features reached good discrimination performance for pathological response prediction.

The exciting breakthroughs seen in the arena of immunotherapy require new imaging approaches to assist in the prediction and assessment of treatment response. Based on response evaluation by imaging, clinicians must decide if they should stop, pause, or continue immunotherapy. Distinguish and assess residual tumor challenged size-based criteria. Metabolic cellular changes are known to precede tumor regression (8). ^18^F-FDG PET/CT has the potential to reflect early changes in the metabolic behavior of malignancies. A recent study found that ^18^F-FDG PET was considered to provide more useful information than CT for immunotherapy assessment in advanced NSCLC patients (7). The lesion consisted of necrotic, fibrous, and residual tumors after neoadjuvant treatment. MPR has been considered a surrogate endpoint for survival prediction in NSCLC. Differentiating between necrotic or fibrous tissue and residual disease is challenging with posttreatment imaging. Metabolic cellular changes are known to precede tumor regression (7, 28), which makes it possible for ^18^F-FDG PET/CT to reflect early changes in the metabolic behavior of malignancies. Posttreatment PET/CT has been proposed to assess treatment response and prognosis prediction, such as the Lugano criterion applied in lymphoma treatment response assessment (29). For NSCLC, Tao et al. (11)found that the degree of pathological regression was negatively correlated with SUL_max_, SUL_peak_, MTV, and TLG of the preoperative PET/CT, despite only limited features analyzed. SUV statistics features had been proven predictive of pathological response in some studies (11, 30). Chen et al. collected 44 patients with stage II-III NSCLC and found delta SUV_max_ was associated with MPR after immunochemotherapy (31). However, heterogeneity existed in the retrospectively enrolled patients. These above studies did not investigate the subtle difference in SUV statistics features. In our study, we compared SUV_max_, SUV_peak_, SUL_max_, SUL_peak_, MTV, and TLG. For MPR prediction, SUV_peak_ and SUL_peak_ performed better than SUV_max_ and SUL_max_, by the iPERCIST recommendation. Considering SUV could be affected by multiple factors, LLR was further analyzed and achieved slightly inferior AUCs and equal accuracies. It could imply the generality of SUV statistics features and the SUV has the advantage for easily available in clinical oncology. Moreover, we tried to simplify the evaluating method of the FDG uptake and applied the Deauville scores applied in lymphoma in this study. The 5PS achieved lower accuracies when applying DS = 4 as a cutoff value, though achieving 100% sensitivity for MPR prediction.

Residual tissue after immunochemotherapy may be different from that of other treatments, therefore reflecting different metabolism patterns and different image features. Tumor cells and influent immune cells both possess high-affinity GLUT1 transporters, thus the high uptake of the lesion after immunotherapy could be a result of a mixture of immune cells and tumor cells and is non-specific. Preoperative PET radiomics might provide additional value to provide useful information on the tumor microenvironment (TME) and heterogeneity to differentiate residual tumor cells and influent immune cells. In our study, SUV statistics features and radiomics features from preoperative PET/CT were analyzed to predict the pathological response. The SUV statistics features are widely used in clinical practice, especially SUV_max_. When collinearity was observed between any SUV statistic features and radiomics features, SUV statistics features were reserved. The End-Uniformity and End-GLDM-LDHGLE were reserved without obvious collinearity. The Uniformity measures the sum of the squares of each intensity value, where a lower uniformity implies greater heterogeneity. The End PET GLDM LDHGLE measures the joint distribution of large dependence with higher gray-level values. LDHGLE is sensitive to large dependences of high gray levels. The multivariate logistic analysis suggested the additional prediction value of the End-GLDM-LDHGLE feature. In addition, the distributions of six delta PET radiomics features were significantly different both in CPR vs. Non-CPR and MPR vs. Non-MPR subgroups. Our study found delta PET features and preoperative PET features had more prognostic value than baseline PET features, similar to other studies (8, 32, 33). We further analyzed delta PET radiomics features. The delta PET features calculated from the forum (delta = [baseline – preoperative]/baseline) reflect the change in PET features and metabolic response after therapy. Due to the limited number of paired PET, the logistic analysis was not performed for delta PET radiomics features. The Delta-GLDM-DN feature was significantly different between different pathological response groups. It measures the change in the uniformity of dependence throughout the image, where the dependency is the number of connected voxels within a distance that is dependent on the center voxel. A higher GLDM-DN value correlates with a greater heterogeneity among dependencies in the image. Tumor heterogeneity might reflect regional tumor cellularity, proliferation, hypoxia, angiogenesis, and factors associated with tumor biological features (33, 34). The features besides SUV statistics could provide additional value and new perspectives for PET interpretation in immunochemotherapy.

Some studies found PET features were associated with clinical biomarkers such as PD-L1 expression and driver gene status (35, 36). A recent study indicated that ^18^F-FDG PET radiomics features could predict strong PD-L1 expression and showed better performance than SUV_max_ (37). The application of immunotherapy in NSCLCs with positive driver genes is unclear (38). Some studies have found significant correlations between the driver gene status and PET features (39, 40). In Tao’s study (11), the relationships between the metabolic parameters and biomarkers such as PD-L1 expression and gene mutations were not analyzed. Nevertheless, our study did not find significant correlations between the baseline PET features and PD-1 expression or driver gene status.

Furthermore, a previous study found the peripheral blood biomarkers had prognostic values of advanced NSCLCs treated with first-line chemo- or immunotherapy and were significantly associated with ^18^F-FDG PET biomarkers, such as bone marrow or spleen-to-liver SUV_max_ ratios (15). Thus, we explored metabolic parameters and other data that rely on peripheral blood sampling in this study. However, no significant associations between peripheral blood biomarkers and pathological responses were found in our study. The data from powerful imaging tools combining the peripheral blood biomarkers including ctDNA need to be investigated further in evaluating responses to neoadjuvant immunochemotherapy.

In this prospective study, all patients performed the first-line treatment strategy that could avoid the bias of the treatment. The adenocarcinoma and squamous carcinoma took up almost equally in this study and this is a study based on the Asian population. In Tao’s study (11), most of the patients with adenocarcinoma were excluded, and the large majority of the patients had squamous cell carcinoma subtype in their trial, which may bias the results in their study. Especially, two patients suffered LELC in our study and both achieved CPR. This type of tumor is with Epstein-Barr virus infection and is preferentially found in nonsmoking Asians. This special subtype could be a good candidate for immunotherapy with its high expression of PD-L1 (defined as a positive tumor proportion score > 5%) (41, 42).

In our study, all images were obtained from two different scanners. Our regular quality control ensured scanning consistency. Moreover, to avoid the bias between different scanners and evaluate the background uptake from different scanners, we compared the uptake of the mediastinal blood pool and liver and its difference between different scanners was not significant.

There are several limitations to our study. Firstly, our study is preliminary and includes a small sample size. The study was performed on the Asian population, using three cycles of toripalimab, nab-paclitaxel or pemetrexed, and carboplatin. The results could be influenced by the small sample size, population, and treatment strategy, thus result in discrepancies with the previously published studies. Further studies including larger numbers of patients from different institutions are necessary to validate these results. Further prospective clinical trials are necessary to confirm the predictive value. Secondly, the follow-up duration was too short to explore survival analysis in detail. We did not evaluate clinical endpoints such as overall survival (OS), as our study focused on MPR. Long-term follow-up is necessary to confirm the prognostic value of ^18^F-FDG PET/CT. Thirdly, not all patients received the baseline ^18^F-FDG PET/CT. Therefore, we cannot investigate the efficacy of PERCIST further. It is needed to include a sufficient number of cases and more closely standardize the imaging criteria of enrolled patients in the future to improve the persuasiveness and to further discuss and validate the results. Fourth, all patients received neoadjuvant immunochemotherapy in our study. However, the same histological changes can also be seen in resected specimens without a history of neoadjuvant therapy and might influence the posttreatment pathological response prediction. Further studies comparing the cohorts with or without neoadjuvant treatment could be performed.

## Conclusion

In this study, we developed a logistic regression model using comprehensive PET features to predict the pathological response after neoadjuvant toripalimab plus chemotherapy treatment in patients with resectable stage III NSCLC. The proposed models based on the post-neoadjuvant-treatment PET features reached good discrimination performance for pathological response prediction. Radiomics features on ^18^F-FDG PET images provide more information that may complement the classical metabolic parameters for response assessment of immunochemotherapy.

## Data availability statement

The original contributions presented in the study are included in the article/**Supplementary Material**. Further inquiries can be directed to the corresponding authors.

## Ethics statement

The studies involving human participants were reviewed and approved by Sun Yat-Sen Cancer Center. Written informed consent for participation was not required for this study in accordance with the national legislation and the institutional requirements.

## Author contributions

Material preparation and data collection were performed by YL, ZZ, HL and LZ. Formal analysis and investigation were performed by YC and XL. The first draft of the manuscript was written by YC and XL. All authors commented on previous versions of the manuscript. All authors contributed to the article and approved the submitted version.

## Funding

This work was supported by the Guangdong Province Natural Science Foundation Fund Project [Grant No. 2022A1515012327].

## Conflict of interest

The authors declare that the research was conducted in the absence of any commercial or financial relationships that could be construed as a potential conflict of interest.

## Publisher’s note

All claims expressed in this article are solely those of the authors and do not necessarily represent those of their affiliated organizations, or those of the publisher, the editors and the reviewers. Any product that may be evaluated in this article, or claim that may be made by its manufacturer, is not guaranteed or endorsed by the publisher.
