# Supplementary material for: Comprehensive 18F-FDG PET-based radiomics in elevating the pathological response to neoadjuvant immunochemotherapy for resectable stage III non-small-cell lung cancer: A pilot study
Source: Front Immunol. 2022 Nov 17;13:994917. doi: 10.3389/fimmu.2022.994917 (PMC9713843; doi:10.3389/fimmu.2022.994917)
Supplement: Supplementary file 1 [file DataSheet_1.docx]

***Supplementary Material 1.***

**Table S1** Summary of PET examination and SUV statistics PET features

| ID | Histology | Baseline | | | | | | | | Preoperative | | | | | | | | Pathologic Response |
| --- | --- | --- | --- | --- | --- | --- | --- | --- | --- | --- | --- | --- | --- | --- | --- | --- | --- | --- |
|  |  | PET | scanner | SUV_max_ | SUV_peak_ | SUL_max_ | SUL_peak_ | MTV | TLG | PET | scanner | SUV_max_ | SUV_peak_ | SUL_max_ | SUL_peak_ | MTV | TLG |  |
| 1 | SQCC | Yes | 2 | 10.90 | 9.12 | 9.54 | 7.98 | 19.87 | 135.79 | Yes | 2 | 7.90 | 4.76 | 6.91 | 4.16 | 1.69 | 7.77 | -100 |
| 2 | ADC | No |  |  |  |  |  |  |  | Yes | 2 | 3.42 | 2.66 | 2.67 | 2.08 | 34.87 | 65.63 | -100 |
| 3 | SQCC | No |  |  |  |  |  |  |  | Yes | 2 | 1.64 | 1.48 | 1.17 | 1.05 | 0.52 | 0.78 | -100 |
| 4 | ADC | Yes | 2 | 13.49 | 11.80 | 10.48 | 9.17 | 19.54 | 172.04 | Yes | 2 | 3.31 | 2.70 | 2.44 | 1.99 | 2.42 | 5.73 | -100 |
| 5 | SQCC | Yes | 2 | 6.41 | 4.42 | 4.38 | 3.02 | 4.21 | 14.59 | Yes | 2 | 2.07 | 1.71 | 1.40 | 1.16 | 0.56 | 0.90 | -100 |
| 6 | ADC | No |  |  |  |  |  |  |  | Yes | 2 | 3.51 | 2.49 | 2.30 | 1.63 | 1.73 | 4.02 | -97 |
| 7 | LELC | No |  |  |  |  |  |  |  | Yes | 2 | 1.64 | 1.43 | 1.12 | 0.98 | 0.33 | 0.51 | -100 |
| 8 | SQCC | No |  |  |  |  |  |  |  | Yes | 2 | 3.98 | 3.27 | 2.87 | 2.36 | 2.39 | 7.57 | -90 |
| 9 | SQCC | No |  |  |  |  |  |  |  | Yes | 2 | 8.32 | 5.18 | 6.34 | 3.95 | 1.76 | 8.51 | -40 |
| 10 | SQCC | Yes | 2 | 10.93 | 7.93 | 8.55 | 6.20 | 8.46 | 52.76 | No |  |  |  |  |  |  |  | -50 |
| 11 | SQCC | No |  |  |  |  |  |  |  | Yes | 2 | 1.53 | 1.44 | 1.21 | 1.13 | 0.66 | 0.65 | -100 |
| 12 | ADC | Yes | 2 | 12.00 | 10.33 | 9.13 | 7.86 | 10.95 | 79.71 | Yes | 2 | 3.72 | 2.62 | 2.85 | 2.00 | 0.93 | 2.76 | -100 |
| 13 | ADC | No |  |  |  |  |  |  |  | Yes | 2 | 4.65 | 3.65 | 3.65 | 2.87 | 4.15 | 12.91 | -30 |
| 14 | ADC | Yes | 2 | 25.37 | 21.77 | 22.13 | 18.99 | 12.87 | 205.48 | Yes | 2 | 16.66 | 12.33 | 14.24 | 10.54 | 2.79 | 29.15 | -55 |
| 15 | LELC | No |  |  |  |  |  |  |  | Yes | 2 | 2.20 | 1.58 | 1.62 | 1.16 | 0.56 | 0.89 | -100 |
| 16 | SQCC | Yes | 1 | 22.02 | 19.20 | 16.06 | 14.01 | 28.92 | 423.96 | Yes | 2 | 2.47 | 2.11 | 1.69 | 1.44 | 0.37 | 0.84 | -100 |
| 17 | SQCC | No |  |  |  |  |  |  |  | Yes | 2 | 1.82 | 1.42 | 1.31 | 1.02 | 20.34 | 19.33 | -98 |
| 18 | ADC | Yes | 1 | 13.77 | 11.59 | 9.87 | 8.31 | 15.71 | 122.95 | Yes | 2 | 9.17 | 7.17 | 6.54 | 5.11 | 15.59 | 78.35 | -10 |
| 19 | ADC | Yes | 2 | 20.34 | 15.54 | 16.73 | 12.78 | 8.26 | 96.12 | Yes | 1 | 9.35 | 5.92 | 7.64 | 4.84 | 2.27 | 12.50 | -92 |
| 20 | SQCC | Yes | 2 | 13.22 | 10.43 | 11.08 | 8.74 | 16.19 | 116.26 | Yes | 2 | 4.20 | 3.64 | 3.31 | 2.87 | 5.74 | 15.05 | -100 |
| 21 | SQCC | Yes | 2 | 18.07 | 15.64 | 13.12 | 11.35 | 37.32 | 402.21 | Yes | 1 | 2.85 | 2.43 | 2.12 | 1.81 | 1.55 | 3.92 | -100 |
| 22 | SQCC | Yes | 2 | 12.95 | 11.53 | 9.77 | 8.70 | 19.08 | 150.99 | Yes | 1 | 13.44 | 9.84 | 10.14 | 7.42 | 3.92 | 29.18 | -35 |
| 23 | ADC | Yes | 1 | 21.85 | 17.82 | 17.36 | 14.16 | 140.27 | 1795.52 | Yes | 2 | 4.71 | 3.77 | 3.74 | 3.00 | 5.64 | 17.06 | -100 |
| 24 | SQCC | No |  |  |  |  |  |  |  | Yes | 1 | 4.16 | 2.75 | 2.97 | 1.96 | 0.90 | 2.84 | -100 |
| 25 | SQCC | No |  |  |  |  |  |  |  | Yes | 2 | 2.82 | 2.15 | 2.22 | 1.70 | 0.27 | 0.70 | -100 |
| 26 | ADC | No |  |  |  |  |  |  |  | Yes | 2 | 9.56 | 7.20 | 6.49 | 4.89 | 3.82 | 20.95 | -50 |
| 27 | ADC | No |  |  |  |  |  |  |  | Yes | 1 | 5.70 | 5.15 | 4.03 | 3.64 | 11.32 | 42.59 | -20 |
| 28 | ADC | No |  |  |  |  |  |  |  | Yes | 1 | 6.30 | 5.38 | 4.78 | 4.08 | 11.64 | 42.30 | -70 |
| 29 | SQCC | No |  |  |  |  |  |  |  | Yes | 1 | 6.08 | 3.29 | 4.86 | 2.63 | 1.03 | 3.55 | -100 |
| 30 | ADC | No |  |  |  |  |  |  |  | Yes | 2 | 6.40 | 4.53 | 4.75 | 3.36 | 7.10 | 23.82 | -75 |

ADC, adenocarcinoma; SQCC, squamous cell cancer; LELC, lymphoid epithelial-like carcinoma.

Scanner 1, uEXPLORER; Scanner 2, Biograph mCT.

**Table S2** Summary of PET metabolic parameters of aorta and liver between two scanners

|  | Biograph mCT | uEXPLORER | *p* value |
| --- | --- | --- | --- |
| Aorta |  |  |  |
| SUV_max_ | 1.56 ± 0.32 | 1.59 ± 0.24 | 0.796 |
| SUV_mean_ | 1.50 ± 0.30 | 1.49 ± 0.22 | 0.765 |
| Liver |  |  |  |
| SUV_max_ | 3.12 ± 0.35 | 2.95 ± 0.23 | 0.228 |
| SUV_mean_ | 2.53 ± 0.32 | 2.57 ± 0.19 | 0.148 |

***Supplementary Material 2.***

**2.1 Detection of the pathologic response of tumor specimens**

Pathological assessment was performed according to the methods described by Cottrell et al (3). Surgical specimens of lung cancer after induction therapy are examined according to the operating guidelines of Hellmann et al., published in Lancet Oncology in 2014 (4). The specific steps are as follows: 1. Measure the maximum diameter (a cm); 2. Take hematoxylin- and eosin-stained slides of at least 1 section per greatest tumor diameter; 3. Calculate the proportion of viable tumor cells, necrosis, stromal tissues and inflammatory cells in each slide; 4. Sum the proportion of viable tumor cells in each slide and take the average value, which is the average proportion of viable tumor cells. If value is less than 10%, the patient is considered achieving MPR. CPR was defined as no viable tumor on all slides of the entire tumor bed.

**2.2 PD-L1 immunohistochemistry**

PD-L1 tumor proportion score (TPS) was assessed using PD-L1 immunohistochemistry assay (22C3 pharmaDx, Code SK006; Dako, Glostrup, Denmark) in formalin-fixed tumor diagnostic samples following the manufacturer’s instructions and international guidelines. Percentage of tumor cells showed membranous PD-L1 expression was recorded.

**2.3 Tumor mutational burden**

For tumor samples underwent tumor mutational burden (TMB) assessment in this trial, library generation and sequencing was performed on NGS platform GENETRON S2000 (Genetron Health, Beijing, China).

***Supplementary Material 3.***

**Table S3** LDH, CRP, dNLR, PLR, and SII of baseline, posttreatment, and delta distributions in the two groups (CPR vs. Non-CPR, MPR vs. Non-MPR).

| Characteristic | CPR | Non-CPR | MPR | Non-MPR |
| --- | --- | --- | --- | --- |
| Baseline |  |  |  |  |
| LDH, *p* value | 0.238 |  | 0.530 |  |
| High | 3 | 0 | 3 | 0 |
| Low | 13 | 12 | 16 | 9 |
| CRP, *p* value | 0.355 |  | 1.000 |  |
| High | 4 | 1 | 4 | 1 |
| Low | 12 | 11 | 15 | 8 |
| dNLR, *p* value | 0.694 |  | 0.431 |  |
| High | 4 | 5 | 5 | 4 |
| Low | 12 | 9 | 15 | 6 |
| PLR, *p* value | 0.722 |  | 0.235 |  |
| High | 9 | 9 | 10 | 8 |
| Low | 7 | 5 | 10 | 2 |
| SII, *p* value | 1.000 |  | 0.690 |  |
| High | 5 | 5 | 6 | 4 |
| Low | 11 | 9 | 14 | 6 |
| Posttreatment |  |  |  |  |
| LDH, *p* value | 0.209 |  | 1.000 |  |
| High | 0 | 2 | 1 | 1 |
| Low | 16 | 12 | 19 | 9 |
| CRP, *p* value | 0.467 |  | 0.333 |  |
| High | 0 | 1 | 0 | 1 |
| Low | 16 | 13 | 20 | 9 |
| dNLR, *p* value | 1.000 |  | 1.000 |  |
| High | 2 | 1 | 2 | 1 |
| Low | 14 | 12 | 18 | 8 |
| PLR, *p* value | 1.000 |  | 1.000 |  |
| High | 5 | 4 | 6 | 3 |
| Low | 11 | 9 | 14 | 6 |
| SII, *p* value | - |  | - |  |
| High | 0 | 0 | 0 | 0 |
| Low | 16 | 13 | 20 | 9 |
| Delta |  |  |  |  |
| LDH, *p* value | 0.403 |  | 0.210 |  |
| Median [IQR] | -0.104(-0.158, 0.028) | -0.109(-0.341, -0.046) | -0.099(-0.158, 0.047) | -0.114(-0.335, -0.082) |
| CRP, *p* value | 0.307 |  | 0.363 |  |
| Median [IQR] | 0.728(0.066, 0.941) | 0.645(-0.357, 0.864) | 0.645(0.066, 0.904) | 0.667(-0.387, 0.836) |
| dNLR, *p* value | 0.405 |  | 0.850 |  |
| Median [IQR] | 0.138(0.071, 0.553) | 0.273(0.215, 0.499) | 0.219(0.086, 0.553) | 0.273(0.215, 0.367) |
| PLR, *p* value | 0.693 |  | 0.278 |  |
| Median [IQR] | 0.204(0.099, 0.522) | 0.261(0.137, 0.414) | 0.187(0.099, 0.461) | 0.354(0.224, 0.521) |
| SII, *p* value | 1.000 |  | 0.925 |  |
| Median [IQR] | 0.554(0.355, 0.765) | 0.616(0.395, 0.760) | 0.554(0.355, 0.774) | 0.616(0.395, 0.719) |

**Table S4** Correlations between the age, gender, histology, baseline clinical stage, or smoking status and LDH, CRP, dNLR, PLR, or SII.

| Characteristic | High LDH | Low LDH | High CRP | Low CRP | High dNLR | Low dNLR | High PLR | Low PLR | High SII | Low SII |
| --- | --- | --- | --- | --- | --- | --- | --- | --- | --- | --- |
| Age, *p* value | 0.145 |  | 0.413 |  | 0.063 |  | 0.095 |  | 0.350 |  |
| median(range) | 68(56-71) | 59(33-68) | 63(56-68) | 59(33-71) | 63(58-71) | 59(33-68) | 62(35-71) | 57(33-67) | 61(52-71) | 59(33-68) |
| Sex, *p* value | 1.000 |  | 0.550 |  | 0.637 |  | 1.000 |  | 0.633 |  |
| Female | 0 | 5 | 0 | 5 | 1 | 5 | 4 | 2 | 1 | 5 |
| Male | 3 | 20 | 5 | 18 | 8 | 16 | 14 | 10 | 9 | 15 |
| Histology, *p* value | 0.556 |  | 1.000 |  | 0.228 |  | 0.460 |  | 0.114 |  |
| ADC | 2 | 9 | 2 | 9 | 6 | 7 | 9 | 4 | 7 | 6 |
| SQCC | 1 | 14 | 3 | 12 | 3 | 12 | 8 | 7 | 3 | 12 |
| Stage, *p* value | 1.000 |  | 1.000 |  | 1.000 |  | 0.709 |  | 0.461 |  |
| IIIA | 2 | 16 | 3 | 15 | 5 | 13 | 10 | 8 | 5 | 13 |
| IIIB | 1 | 9 | 2 | 8 | 4 | 8 | 8 | 4 | 5 | 7 |
| Smoking, *p* value | 0.551 |  | 0.290 |  | 0.666 |  | 0.419 |  | 0.682 |  |
| No | 0 | 7 | 0 | 7 | 3 | 5 | 6 | 2 | 2 | 6 |
| Yes | 3 | 18 | 5 | 16 | 6 | 16 | 12 | 10 | 8 | 14 |

**Table S5** Correlations between baseline BLR, SLR, or PET features and LDH, CRP, dNLR, PLR, or SII.

| Characteristic | High LDH | Low LDH | High CRP | Low CRP | High dNLR | Low dNLR | High PLR | Low PLR | High SII | Low SII |
| --- | --- | --- | --- | --- | --- | --- | --- | --- | --- | --- |
| BLR, *p* value | 0.769 |  | 0.811 |  | 0.622 |  | 0.940 |  | 0.354 |  |
| Median [IQR] | 0.838(0.822-0.854) | 0.839(0.795-0.945) | 0.839(0.809-1.156) | 0.845(0.807-0.912) | 0.839(0.805-1.216) | 0.845(0.8-0.89) | 0.839(0.805-0.968) | 0.85(0.806-0.89) | 0.805(0.779-0.839) | 0.875(0.818-0.934) |
| SLR, *p* value | 0.641 |  | 0.469 |  | 0.622 |  | 0.148 |  | 0.524 |  |
| Median [IQR] | 0.714(0.678-0.75) | 0.764(0.706-0.82) | 0.777(0.762-0.82) | 0.74(0.694-0.782) | 0.777(0.746-0.862) | 0.74(0.696-0.773) | 0.777(0.746-0.862) | 0.704(0.68-0.729) | 0.777(0.746-0.862) | 0.74(0.696-0.773) |
| SUV_max_, *p* value | 0.308 |  | 0.811 |  | 0.724 |  | 0.604 |  | 0.284 |  |
| Median [IQR] | 19.961(19.014-20.907) | 13.222(11.462-17.053) | 11.997(11.462-17.006) | 13.628(13.021-19.77) | 13.768(11.997-21.853) | 13.354(12.44-18.635) | 13.768(11.997-21.853) | 13.354(11.518-15.2) | 21.853(11.997-22.015) | 13.354(12.44-14.843) |
| TLG, *p* value | 0.051 |  | 0.573 |  | 0.943 |  | 0.199 |  | 0.524 |  |
| Median [IQR] | 1098.863(750.536-1447.19) | 122.952(87.91-161.517) | 79.706(66.232-251.833) | 143.39(117.934-197.123) | 122.952(79.706-423.959) | 143.39(111.225-180.401) | 150.994(122.952-402.209) | 106.189(75.734-130.207) | 205.484(79.706-423.959) | 129.368(111.225-156.255) |

**Table S6** Correlations between posttreatment BLR, SLR, or PET features and LDH, CRP, dNLR, PLR, or SII.

| Characteristic | High LDH | Low LDH | High CRP | Low CRP | High dNLR | Low dNLR | High PLR | Low PLR | High SII | Low SII |
| --- | --- | --- | --- | --- | --- | --- | --- | --- | --- | --- |
| BLR, *p* value | 0.315 |  | 0.552 |  | 0.219 |  | 0.735 |  | - |  |
| Median [IQR] | 1.098(1.086-1.11) | 0.846(0.702-1.097) | 0.708(0.708-0.708) | 0.892(0.735-1.11) | 1.074(1.062-1.117) | 0.846(0.695-1.105) | 1.051(0.695-1.122) | 0.871(0.763-1.09) | - | 0.892(0.735-1.11) |
| SLR, *p* value | 1.000 |  | 0.069 |  | 0.248 |  | 0.885 |  | - |  |
| Median [IQR] | 0.751(0.732-0.769) | 0.749(0.632-0.815) | 0.899(0.899-0.899) | 0.747(0.632-0.799) | 0.808(0.761-0.839) | 0.744(0.632-0.792) | 0.777(0.628-0.823) | 0.744(0.669-0.794) | - | 0.747(0.632-0.799) |
| SUV_max_, *p* value | 0.148 |  | 0.207 |  | 0.433 |  | 0.595 |  | - |  |
| Median [IQR] | 7.872(7.135-8.609) | 3.984(2.644-6.189) | 9.56(9.56-9.56) | 4.074(2.729-6.326) | 2.473(2.054-4.435) | 4.163(2.852-6.302) | 5.695(2.852-7.903) | 3.984(2.644-5.393) | - | 4.074(2.729-6.326) |
| TLG, *p* value | 0.443 |  | 0.552 |  | 0.280 |  | 0.962 |  | - |  |
| Median [IQR] | 18.156(15.326-20.985) | 7.569(1.829-20.138) | 20.948(20.948-20.948) | 7.669(2.296-20.449) | 0.841(0.673-12.328) | 7.769(2.844-19.327) | 7.769(2.762-29.184) | 7.569(1.87-18.195) | - | 7.669(2.296-20.449) |
| Uniformity, *p* value | 0.355 |  | 0.828 |  | 0.125 |  | 0.595 |  | - |  |
| Median [IQR] | 0.106(0.086-0.126) | 0.149(0.101-0.265) | 0.134(0.134-0.134) | 0.147(0.096-0.251) | 0.52(0.333-0.529) | 0.141(0.091-0.218) | 0.104(0.086-0.291) | 0.149(0.112-0.215) | - | 0.147(0.096-0.251) |
| GLDM-LDHGLE, *p* value | 0.833 |  | 0.483 |  | 0.090 |  | 0.847 |  | - |  |
| Median [IQR] | 504.993(458.507-551.478) | 406.306(119.923-782.996) | 783.159(783.159-783.159) | 409.164(120.962-764.427) | 67.455(65.427-239.738) | 537.296(126.421-782.833) | 587.051(169.032-718.023) | 406.306(119.923-770.562) | - | 409.164(120.962-764.427) |

***Supplementary Material 4.***

The constructed multivariate logistic models for CPR and MPR prediction.

CRP prediction model

SUV_max_ model: $ln\frac{p}{1-p}=0.555\times{SUV}_{max}+0.003\times End\_GLDM\_LDHGLE-4.468$

SUV_peak_ model: $ln\frac{p}{1-p}=0.979\times{SUV}_{peak}+0.003\times End\_GLDM\_LDHGLE-5.066$

SUL_max_ model: $ln\frac{p}{1-p}=0.558\times{SUL}_{max}+0.003\times End\_GLDM\_LDHGLE-3.873$

SUL_peak_ model: $ln\frac{p}{1-p}=0.997\times{SUL}_{peak}+0.003\times End\_GLDM\_LDHGLE-4.345$

MPR prediction model

SUV_max_ model: $ln\frac{p}{1-p}=0.678\times{SUV}_{max}+0.060\times TLG-5.889$

SUV_peak_ model: $ln\frac{p}{1-p}=1.514\times{SUV}_{peak}-7.083$

SUL_max_ model: $ln\frac{p}{1-p}=0.700\times{SUL}_{max}+0.061\times TLG-5.056$

SUL_peak_ model: $ln\frac{p}{1-p}=1.608\times{SUL}_{peak}-5.877$
